# Pharmacokinetic drug-drug interaction between the COVID-19 3CL protease inhibitor GST-HG171 and itraconazole in healthy subjects

**DOI:** 10.1128/aac.00285-26

**Published:** 2026-05-11

**Authors:** Xiaojiao Li, Bing Li, Meng Wang, Jing Wang, Jing Lu, John Mao, Tianxiang Zhang, Yanan Tang, Wenhao Yan, Chuanjing Li, George Zhang, Yanhua Ding

**Affiliations:** 1 The First Hospital of Jilin University, Phase I Clinical Research Center 117971, Jilin, China; 2 Fujian Akeylink Biotechnology Co., Ltd., Fuzhou, Fujian, China; Chinese Academy of Medical Sciences & Peking Union Medical College, Beijing, China

**Keywords:** drug-drug interactions, pharmacokinetics, GST-HG171, itraconazole, COVID-19

## Abstract

GST-HG171 (also known as atilotrelvir) is a novel, orally administered 3CL protease inhibitor approved in China for treating mild-to-moderate COVID-19. In this study, we assessed the drug-drug interaction between GST-HG171 (boosted with ritonavir) and itraconazole (a strong CYP3A inhibitor) in healthy Chinese adult volunteers. A total of 12 healthy subjects were enrolled in this two-period study. In period 1, all subjects received 150 mg GST-HG171/100 mg ritonavir twice daily (BID) on Days 1–3 (with a single dose on Day 3); after a 5-day washout period, subjects proceeded to period 2. In period 2, subjects orally took 200 mg itraconazole once daily (QD) on Days 8–16. On Days 12–14, subjects were co-administered 150 mg GST-HG171/100 mg ritonavir (BID, with a single dose on Day 14) and itraconazole. Serial blood samples were collected after administration of GST-HG171/ritonavir alone and co-administration of itraconazole for the measurement of pharmacokinetic parameters. Co-administration of itraconazole with GST-HG171/ritonavir resulted in a mild increase in the *C*_max_ of GST-HG171 and ritonavir by approximately 8% and 28%, respectively, while systemic exposure (area under the plasma concentration-time curve [AUC]) was elevated by approximately 45% and 33%, respectively. There was no apparent increase in the incidence or severity of AEs during the co-administration period. In conclusion, co-administration of itraconazole, a strong CYP3A4 inhibitor, with GST-HG171/ritonavir resulted in a moderate increase in exposure of GST-HG171 and mild increase in exposure of ritonavir, and it had an acceptable safety profile and was well tolerated in this study.

**CLINICAL TRIALS:**

This study is registered with ClinicalTrials.gov as NCT06087055.

## INTRODUCTION

The global pandemic of coronavirus disease 2019 (COVID-19), caused by severe acute respiratory syndrome coronavirus 2 (SARS-CoV-2), has posed an unprecedented challenge to public health systems worldwide since its emergence in late 2019 (1, 2). Although the pathogenicity of recent Omicron subvariants (e.g., BA.2.86, XBB.1.5) has decreased compared to earlier strains (e.g., Alpha, Beta, Delta), their markedly enhanced transmissibility and immune evasion capabilities have led to widespread population susceptibility (3, 4). Moreover, as new variants continue to emerge, the potential for long-term consequences remains largely unknown.

The urgent need for effective therapeutics has spurred the development of antiviral strategies targeting both viral proteins and host factors. Among these, the viral main protease (Mpro or 3C-like protease, 3CLpro) represents a particularly attractive drug target due to its indispensable role in processing viral polyproteins, its high conservation across SARS-CoV-2 variants, and its low risk for human off-target effects (5, 6). Nirmatrelvir, a component of the first-in-class oral antiviral regimen nirmatrelvir-ritonavir (Paxlovid), is a potent 3CL protease inhibitor that has demonstrated efficacy in reducing hospitalization and mortality in high-risk patients. However, the potential for viral resistance under selective pressure, coupled with affordability and accessibility issues for a substantial number of patients globally, underscores the persistent demand for the development of novel, safe, effective, and affordable oral antivirals (7–10).

GST-HG171 (also known as atilotrelvir) is a novel, orally administered 3CL protease inhibitor approved in China in 2023 for treating mild-to-moderate COVID-19 (11). Preclinical and clinical studies have demonstrated its superior potency compared to nirmatrelvir. In preclinical cellular assays, GST-HG171 exhibited broad-spectrum activity against a range of SARS-CoV-2 variants, including wild-type, Beta, Delta, and Omicron sublineages, with 5- to 10-fold higher activity than those of nirmatrelvir (11). In phase I clinical studies among healthy human volunteers, GST-HG171, co-administered with the pharmacokinetic (PK) enhancer ritonavir (a CYP3A4 inhibitor), showed favorable PK characteristics and an excellent safety profile. Population PK models indicate that the clinical efficacy of GST-HG171 (150 mg) plus ritonavir (100 mg) may be comparable or superior to that of nirmatrelvir/ritonavir (300/100 mg) (12). This dosing regimen (GST-HG171 150 mg/ritonavir 100 mg) was subsequently selected and has demonstrated excellent efficacy and safety in pivotal phase II/III clinical trials, compared with a placebo regimen in overall symptom recovery for COVID-19 caused by emerging Omicron variants, including the most recent XBB subtypes (13).

As GST-HG171 is primarily metabolized by cytochrome P450 3A4 (CYP3A4), its co-administration with ritonavir can boost its systemic exposure by three- to sixfold (11). Moreover, CYP3A4 is also the primary enzyme for ritonavir metabolism (14). Itraconazole is a strong CYP3A4 inhibitor, which has been widely used in drug-drug interaction (DDI) studies (15). To investigate the potential for metabolic interactions, this study assessed the DDI between GST-HG171 (including the PK enhancer ritonavir) and itraconazole in healthy Chinese adult volunteers, along with the safety profiles during co-administration.

## MATERIALS AND METHODS

### Study design

**Fig 1:**
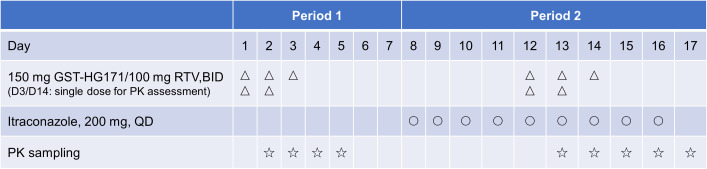
Design of the drug-drug interaction study between GST-HG171/ritonavir and itraconazole.

This study was an open-label, single-center drug-drug interaction (DDI) investigation conducted at the Phase I Clinical Research Center of Jilin University First Affiliated Hospital (Changchun City, China) between 12 May 2023 and 5 June 2023. A total of 12 healthy subjects were enrolled to evaluate the pharmacokinetic profiles and tolerability of GST-HG171 tablets co-administered with ritonavir in the presence of itraconazole capsules. The study design is summarized in Fig. 1. In period 1, all subjects received 150 mg GST-HG171/100 mg ritonavir twice daily (BID) under fasting conditions on Days 1–3 (with a single dose on Day 3), followed by a standard breakfast 2 h post-dose. After a 5-day washout period, subjects proceeded to period 2. During this period, subjects orally took 200 mg itraconazole once daily (QD) approximately 10 min after starting breakfast on Days 8–16. On Days 12–14, they concurrently received 150 mg GST-HG171/100 mg ritonavir (BID) under fasting conditions (with a single dose on Day 14), followed by a standard breakfast 2 h later and itraconazole approximately 10 min after the start of breakfast.

### Subjects

Eligible subjects were Chinese males or females aged 18 to 50 years, minimum weight of 50 kg for males and 45 kg for females and a body mass index (BMI) ranging from 18 to 28 kg/m², with a commitment to effective contraception during the study and for 6 months after the last administration of the study drug and absence of clinically significant abnormalities in physical examination or vital signs.

The main exclusion criteria include the following: blood donation or significant blood loss exceeding 400 mL within 3 months before screening; use of medications affecting metabolic enzymes within 28 days before screening or during the study; participation in other studies within 3 months prior to screening; recent use of prescription drugs or herbal medicines within 14 days before screening; and any clinically significant findings from physical examination, vital signs, laboratory tests, 12-lead ECG, or abdominal ultrasound.

### PK analysis

PK blood samples (approximately 4 mL each) were collected into K2EDTA vacuum tubes at specified time points. On Day 2 and Day 13, samples were collected within 30 min before both scheduled doses of GST-HG171/ritonavir. On Day 3, a pre-dose sample was collected within 30 min, followed by post-dose samples at 0.25, 0.5, 0.75, 1, 1.25, 1.5, 2, 3, 4, 6, 8, 12, 24, and 48 h. On Day 14, a pre-dose sample was collected within 30 min, with subsequent collections at 0.25, 0.5, 0.75, 1, 1.25, 1.5, 2, 3, 4, 6, 8, 12, 24, 48, and 72 h post-dose. Following collection, all samples were centrifuged at 1,500 × g for 10 min under 2–8°C; then, the plasma samples were subsequently stored at –80°C until analysis.

The bioanalysis of GST-HG171 and ritonavir in plasma was performed using a validated liquid chromatography-tandem mass spectrometry (LC-MS/MS) method. The calibration curve for GST-HG171 was linear over the concentration range of 10 to 10,000 ng/mL, with an LLOQ of 10 ng/mL. The calibration curve for ritonavir was linear over the concentration range of 10 to 1,000 ng/mL, with an LLOQ of 10 ng/mL. For quality control samples, both intra- and inter-batch precision and accuracy met the pre-defined acceptance criteria for bioanalytical methods.

### Safety assessments

Safety assessments included physical examinations, vital signs, electrocardiograms (ECG), laboratory tests, and adverse events (AEs). The causality of each AE to the study drug was evaluated, and its severity was graded according to the Common Terminology Criteria for Adverse Events (CTCAE), version 5.0.

### Statistical analysis

The PK parameters of GST-HG171 and ritonavir were estimated using a non-compartmental model with WinNonlin software (version 8.3). A mixed-effects model was applied to assess the drug-drug interaction effect on the logarithmically transformed *C*_max_, area under the plasma concentration-time curve (AUC_0-*t*_), and AUC_0-∞_. The geometric mean ratios (GMRs) (with/without co-administration of itraconazole) and their corresponding 90% confidence intervals (CIs) were calculated for each PK parameter. Statistical analysis was performed using SAS version 9.4 (SAS Institute Inc.) software.

## RESULTS

A total of 35 subjects were screened, of whom 12 were enrolled and all completed the study. The enrolled cohort consisted of 5 males and 7 females, with a mean (±standard deviation [SD]) age of 39.33 ± 6.51 years, height of 162.50 ± 7.49 cm, body weight of 62.95 ± 7.56 kg, and BMI of 23.75 ± 2.26 kg/m². Detailed demographic characteristics are presented in Table 1.

**TABLE 1:** Baseline demographic characteristics of study subjects^*a*^

Characteristic	Total (*N* = 12)
Age, yr	39.3 ± 6.51
Gender, *n* (%)	
Male	5 (41.7%)
Female	7 (58.3%)
Height, cm	162.5 ± 7.49
Weight, kg	63.0 ± 7.56
BMI, kg/m^2^	23.8 ± 2.26

^*a*^ Data are presented as mean ± SD unless otherwise indicated.

### PK results

**Fig 2:**
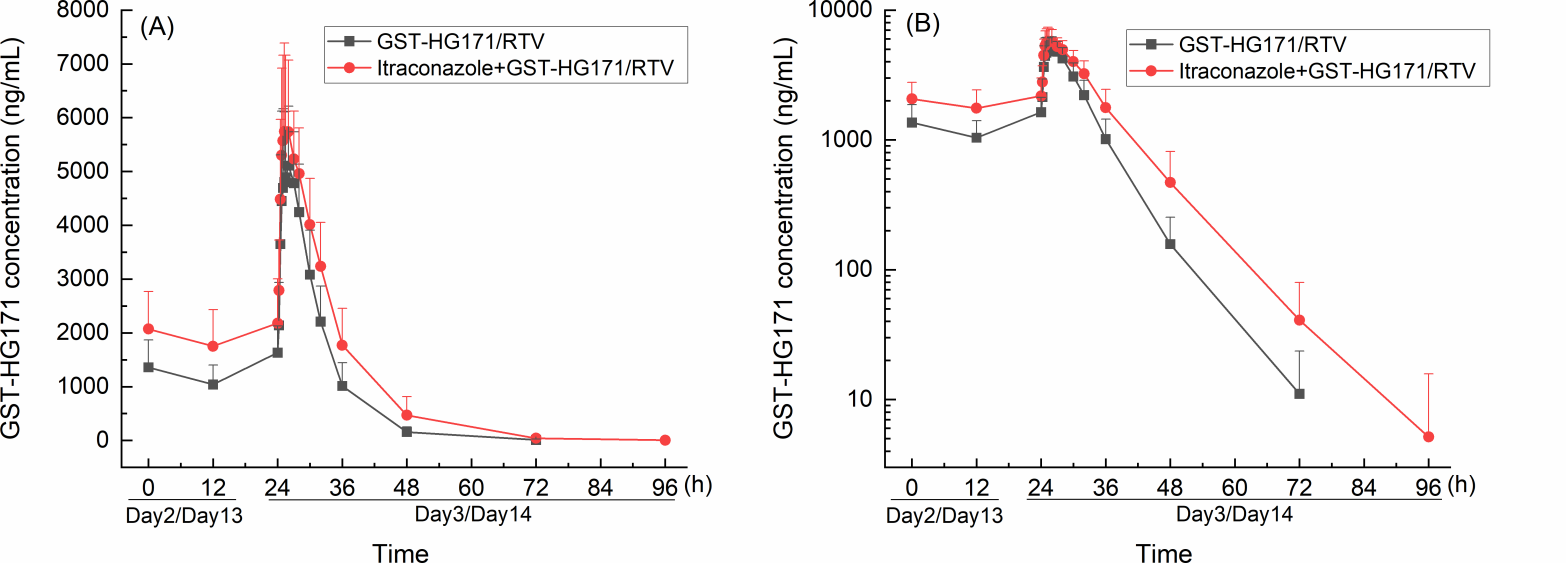
Plasma concentration-time curves of GST-HG171 in GST-HG171/ritonavir monotherapy and co-administration with itraconazole. Mean (+SD) plasma concentration profiles were plotted with linear scale (**A**) and semi-log scale (**B**).

**Fig 3:**
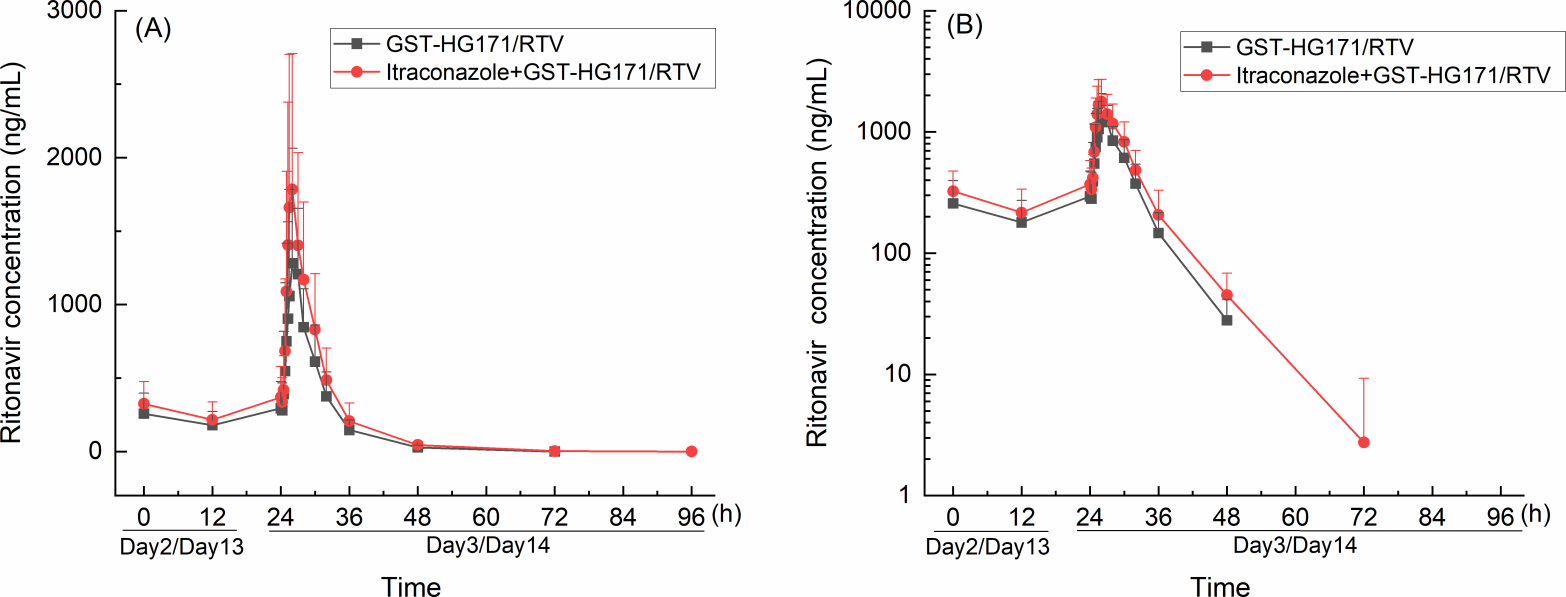
Plasma concentration-time curves of ritonavir in GST-HG171/ritonavir monotherapy and co-administration with itraconazole. Mean (+SD) plasma concentration profiles were plotted with linear scale (**A**) and semi-log scale (**B**).

The plasma concentration-time curves of GST-HG171 and ritonavir in plasma are presented in Fig. 2 and 3. The PK parameters, GMRs, and 90% CIs are detailed in Tables 2 and 3.

**TABLE 2:** Pharmacokinetic parameters of GST-HG171 and ritonavir in GST-HG171/ritonavir monotherapy and co-administration with itraconazole^*a*^

Therapy	PK parameter (unit)	GST-HG171/ritonavir (*N* = 12)	Itraconazole + GST-HG171/ritonavir (*N* = 12)
GST-HG171	*C*_max_ (ng/mL)	5,748.80 ± 1,066.62 (18.55)	6,223.81 ± 1,196.93 (19.23)
	AUC_0-*t*_ (ng·h/mL)	45,120.83 ± 11,319.05 (25.09)	6,6411.14 ± 20,625.66 (31.06)
	AUC_0-∞_ (ng·h/mL)	45,559.24 ± 11,607.99 (25.48)	6,6702.59 ± 20,609.36 (30.90)
	*t*_1/2_ (h)	4.71 ± 1.22 (25.85)	6.20 ± 1.25 (20.13)
	*T*_max_ (h)	1.75 (0.7506, 3.0011)	1.25 (0.5003, 3.0011)
	CL/F (mL/h)	3,504.68 ± 943.19 (26.91)	2,459.79 ± 796.90 (32.40)
	V_z_/F (mL)	2,3149.93 ± 6,614.00 (28.57)	21,077.31 ± 4,555.97 (21.62)
Ritonavir	*C*_max_ (ng/mL)	1,439.58 ± 684.74 (47.57)	1,883.72 ± 900.94 (47.83)
	AUC_0-*t*_ (ng·h/mL)	8,280.67 ± 3,509.34 (42.38)	11,397.95 ± 5,747.06 (50.42)
	AUC_0-∞_ (ng·h/mL)	8,449.27 ± 3,574.10 (42.30)	11,617.58 ± 5,755.22 (49.54)
	*t*_1/2_ (h)	4.15 ± 0.34 (8.21)	4.80 ± 1.15 (23.98)
	*T*_max_ (h)	2.50 (2.0008, 3.0011)	2.00 (1.5003, 3.0011)
	CL/F (mL/h)	13,521.57 ± 4,567.98 (33.78)	10,424.98 ± 4,313.15 (41.37)
	V_z_/F (mL)	81,576.99 ± 29,946.58 (36.71)	68,339.48 ± 22,923.68 (33.54)

^*a*^ All parameters except *T*_max_ are presented as mean ± SD (CV%). *T*_max_ is expressed as the median (minimum, maximum).

**TABLE 3:** Comparison of pharmacokinetic parameters of GST-HG171 and ritonavir using point estimate on geometric mean in GST-HG171/ritonavir monotherapy and co-administration with itraconazole

Treatment	PK parameter (unit)	Geometric mean		Geometric mean ratio		Intra-individual coefficient of variation (CV%)
		Itraconazole + GST-HG171/ritonavir (*N* = 12)	GST-HG171/ritonavir (*N* = 12)	Itraconazole +GST-HG171/ritonavir vs. GST-HG171/ritonavir (%)	90% CI (%)	
GST-HG171	*C*_max_ (ng/mL)	6,109.19	5,655.86	108.02	93.96–124.17	20.09
	AUC_0-*t*_ (ng·h/mL)	63,501.04	43,795.7	144.99	118.45–177.49	29.46
	AUC_0-∞_ (ng·h/mL)	63,821.33	44,182.15	144.45	117.99–176.84	29.47
Ritonavir	*C*_max_ (ng/mL)	1,691.29	1,321.18	128.01	92.97–176.26	48.1
	AUC_0-*t*_ (ng·h/mL)	10,263.83	7,704.32	133.22	98.72–179.79	44.79
	AUC_0-∞_ (ng·h/mL)	10,493.12	7,864.84	133.42	99.13–179.56	44.34

Co-administration of itraconazole with GST-HG171/ritonavir slightly increased the *C*_max_ of GST-HG171 by approximately 8% (GMR: 108.20%; 90% CI: 93.96–124.17), elevated its systemic exposure by approximately 45% (AUC_0–*t*_ GMR: 144.99%, 90% CI: 118.45–177.49; AUC_0–∞_ GMR: 144.45%, 90% CI: 117.99–176.84), and prolonged its mean *t*_1/2_ by approximately 1.5 h.

Co-administration of itraconazole with GST-HG171/ritonavir mildly increased the *C*_max_ of ritonavir by approximately 28% (GMR: 128.01%; 90% CI: 92.97–176.26), elevated its systemic exposure by approximately 33% (AUC_0–*t*_ GMR: 133.22%, 90% CI: 98.72–179.79; AUC_0–∞_ GMR: 133.42%, 90% CI: (99.13–179.56)), and no significant prolongation of the *t*_1/2_ was observed.

### Safety

All 12 enrolled subjects completed the study drug administration, and both the GST-HG171 tablets/ritonavir regimen alone and co-administration with itraconazole demonstrated favorable safety and tolerability. Over the two treatment periods, a total of nine subjects (75.0%, 9/12) experienced AEs (Table 4). Of these, AEs related to GST-HG171/ritonavir were reported in eight subjects (66.7%, 8/12), and AEs related to itraconazole were reported in five subjects (41.7%, 5/12). The majority of AEs were mild (CTCAE Grade 1). Grade 2 AEs occurred at an incidence of 25.0% (3/12). No Grade 3 or higher AEs, serious adverse events (SAEs), or AEs leading to drug discontinuation, study withdrawal, or death were reported. All AEs resolved completely without sequelae and did not require any therapeutic intervention.

**TABLE 4:** Treatment-emergent adverse event during the drug-drug interaction study between GST-HG171/ritonavir and itraconazole^*a*^

Adverse event(s)	Period 1 (*N* = 12)	Period 2 (*N* = 12)			Total (*N* = 12)
	GST-HG171/ritonavir	Itraconazole	Itraconazole + GST-HG171/ritonavir	Total	
All AEs	6 (50.0)	3 (25.0)	4 (33.3)	5 (41.7)	9 (75.0)
All GST-HG171/ritonavir-related AEs	6 (50.0)	1 (8.3)	3 (25.0)	3 (25.0)	8 (66.7)
All Itraconazole-related AEs	2 (16.7)	3 (25.0)	3 (25.0)	4 (33.3)	5 (41.7)
Serum triglyceride increased	3 (25.0)	1 (8.3)	0 (0)	1 (8.3)	4 (33.3)
Serum creatinine increased	0 (0)	0 (0)	2 (16.7)	2 (16.7)	2 (16.7)
Serum creatine phosphokinase increased	0 (0)	0 (0)	1 (8.3)	1 (8.3)	1 (8.3)
Serum albumin decreased	1 (8.3)	0 (0)	0 (0)	0 (0)	1 (8.3)
WBC count decreased	0 (0)	1 (8.3)	0 (0)	1 (8.3)	1 (8.3)
Lymphocyte count decreased	0 (0)	0 (0)	1 (8.3)	1 (8.3)	1 (8.3)
Neutrophil count decreased	0 (0)	1 (8.3)	0 (0)	1 (8.3)	1 (8.3)
Rash	0 (0)	2 (16.7)	0 (0)	2 (16.7)	2 (16.7)
Pruritus	0 (0)	1 (8.3)	0 (0)	1 (8.3)	1 (8.3)
Anemia	0 (0)	0 (0)	1 (8.3)	1 (8.3)	1 (8.3)
Nausea	1 (8.3)	0 (0)	0 (0)	0 (0)	1 (8.3)
Myalgia	0 (0)	0 (0)	1 (8.3)	1 (8.3)	1 (8.3)
Hypertriglyceridemia	1 (8.3)	0 (0)	0 (0)	0 (0)	1 (8.3)

^*a*^ Data are presented as *n* (%).

During GST-HG171/ritonavir treatment alone in period 1, AEs were reported in six subjects (50.0%, 6/12), all of which were considered related to GST-HG171/ritonavir. Among these, serum albumin decreased and hypertriglyceridemia persisted from period 1 through the end of period 2 and were assessed as potentially related to both GST-HG171/ritonavir and itraconazole. The only drug-related AE reported in two or more subjects was serum triglyceride increased, occurring in 25.0% (3/12) of participants.

During the itraconazole-alone administration in period 2, AEs were reported in three subjects (25.0%, 3/12), all of which were assessed as related to itraconazole. Among these AEs, decreased WBC and neutrophil counts, which emerged during the itraconazole-alone administration phase and persisted into the subsequent triple-drug combination phase, were considered potentially related to GST-HG171/ritonavir, as well as itraconazole. Rash was the only drug-related AE reported in two or more subjects, with an incidence of 16.7% (2/12).

During the combined administration of GST-HG171/ritonavir and itraconazole in period 2, AEs were reported in four subjects (33.3%, 4/12). Among these, AEs in three subjects (25.0%, 3/12) were assessed as potentially related to both GST-HG171/ritonavir and itraconazole. One subject experienced increased serum creatine phosphokinase and myalgia, which were judged as possibly unrelated and definitely unrelated to the study drugs, respectively. The only drug-related AE reported in two or more subjects was increased serum creatinine (16.7%, 2/12).

Compared with GST-HG171/ritonavir alone, the incidence and severity of AEs did not increase with GST-HG171/ritonavir + itraconazole combination. The results demonstrate that both the GST-HG171/ritonavir alone and the three-drug combination with itraconazole exhibited favorable safety and tolerability profiles in healthy subjects.

## DISCUSSION

This study assessed the potential clinical implications of drug interactions involving GST-HG171 (including the PK enhancer ritonavir) and the CYP3A4 inhibitor itraconazole. It has been reported that CYP3A4 serves as the primary metabolizing enzyme for both GST-HG171 and ritonavir (11, 14). Since itraconazole is a strong CYP3A4 inhibitor, concomitant administration may lead to increased systemic concentrations and overall exposure of GST-HG171 and ritonavir (15).

As demonstrated in a previous Phase I study, the exposure (*C*_max_ and AUC) of GST-HG171 was similar between the fasting and fed conditions (12). Moreover, according to the ritonavir drug label, following the administration of 100 mg ritonavir, the *C*_max_ and AUC were slightly decreased (~20%) under fed condition compared to fasting condition (14). Therefore, the fasting condition was selected for GST-HG171 and ritonavir. Since itraconazole should be taken with food to maximize its oral bioavailability, the fed condition was selected accordingly (16). In addition, multiple studies indicate that administration of 200 mg of itraconazole for 4 days is sufficient to achieve steady-state inhibition of the CYP3A4 enzyme (15, 17); therefore, in this study, itraconazole 200 mg once daily was administered from Day 8 to Day 16, with co-administration with GST-HG171/ritonavir on Day 12.

Co-administration of itraconazole with GST-HG171/ritonavir resulted in a mild increase in the *C*_max_ of GST-HG171 and ritonavir by approximately 8% and 28%, respectively, while systemic exposure (AUC) was elevated by approximately 45% and 33%, respectively. In this study, the systemic exposure of GST-HG171 following co-administration of 200 mg itraconazole and 150 mg GST-HG171/100 mg ritonavir was comparable to that observed in a multiple-dose clinical trial in healthy subjects receiving 300 mg GST-HG171 combined with 100 mg ritonavir (12). Furthermore, literature indicates that the clinical dose of ritonavir for HIV treatment is 600 mg twice daily (14, 18). The exposure at this therapeutic dose is substantially higher than the ritonavir exposure observed in the present study under the triple-drug combination regimen. In addition, ritonavir, when used as a pharmacokinetic booster, achieves maximum inhibition of CYP3A at a dose of 100 mg twice daily, even if the dose is increased (19). In this study, after co-administration with another CYP3A4 inhibitor, itraconazole, only a mild-to-moderate increase in the exposure of ritonavir and GST-HG171 was observed, which may reflect either a ceiling effect of CYP3A4 inhibition or the nonlinear pharmacokinetics of ritonavir because of auto-inhibition (20).

Although GST-HG171 and ritonavir exposure was increased by approximately 40% during co-administration of itraconazole, there was no apparent increase in the incidence or severity of AEs. The safety profile was favorable both with and without itraconazole co-administration. The majority of AEs were mild, and no Grade 3 or higher AEs, SAEs, or AEs leading to drug discontinuation were reported. Nevertheless, due to the limited sample size, the safety profile of the triple-drug combination, especially with respect to rare AEs, requires further evaluation in a larger population.

### Conclusion

Co-administration of itraconazole, a strong CYP3A4 inhibitor, with GST-HG171/ritonavir resulted in a moderate increase in exposure of GST-HG171 and a mild increase in exposure of ritonavir and demonstrated an acceptable safety profile and good tolerability in this study.
